# Microtubules in Influenza Virus Entry and Egress

**DOI:** 10.3390/v12010117

**Published:** 2020-01-17

**Authors:** Caitlin Simpson, Yohei Yamauchi

**Affiliations:** School of Cellular and Molecular Medicine, University of Bristol, Bristol BS8 1TD, UK; cs0513@bristol.ac.uk

**Keywords:** influenza virus, cytoskeleton, microtubules, infection biology, endocytosis, aggresome processing, histone deacetylase, uncoating

## Abstract

Influenza viruses are respiratory pathogens that represent a significant threat to public health, despite the large-scale implementation of vaccination programs. It is necessary to understand the detailed and complex interactions between influenza virus and its host cells in order to identify successful strategies for therapeutic intervention. During viral entry, the cellular microenvironment presents invading pathogens with a series of obstacles that must be overcome to infect permissive cells. Influenza hijacks numerous host cell proteins and associated biological pathways during its journey into the cell, responding to environmental cues in order to successfully replicate. The cellular cytoskeleton and its constituent microtubules represent a heavily exploited network during viral infection. Cytoskeletal filaments provide a dynamic scaffold for subcellular viral trafficking, as well as virus-host interactions with cellular machineries that are essential for efficient uncoating, replication, and egress. In addition, influenza virus infection results in structural changes in the microtubule network, which itself has consequences for viral replication. Microtubules, their functional roles in normal cell biology, and their exploitation by influenza viruses will be the focus of this review.

## 1. Microtubules: Structure, Function and Organisation

The cellular cytoskeleton represents a complex and dynamic network of interacting protein filaments with multiple roles in the biological functioning of cells. Structurally, the cytoskeleton is primarily composed of three major types of protein filaments: actin filaments, intermediate filaments, and microtubules. These proteins function in concert to regulate numerous aspects of cell biology, such as cell topology and spatial arrangement of cellular constituents, cell motility and division during mitosis and meiosis, and regulation of the intracellular transport of a wide array of protein cargoes.

Microtubules comprise a class of cytoskeletal proteins that serve as regulators of a wide variety of biological processes. With functions in regulating cell polarity, cell division-associated chromosome segregation, and intracellular cargo transport, the functional roles of microtubules are wide-ranging [1,2]. As important structural components of specialised cellular features such as cilia and flagella in some cell types, microtubules also serve to establish normal cell morphology.

**Figure 1:**
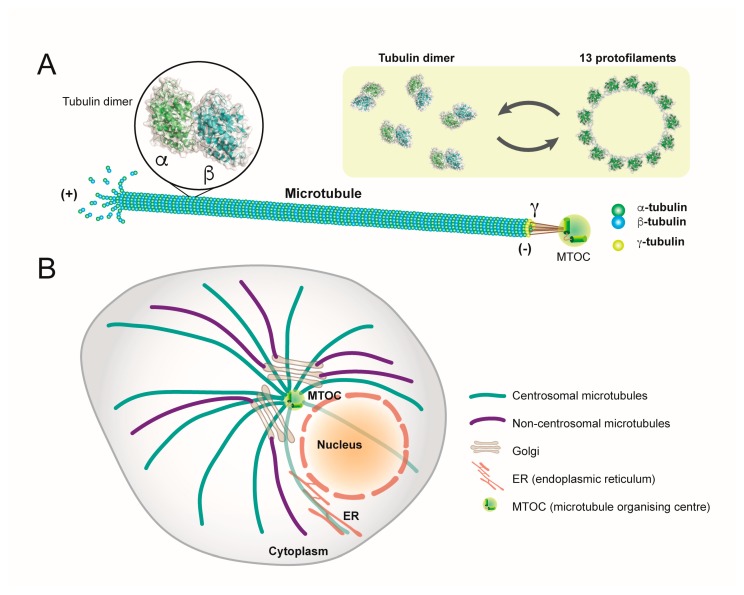
Structure and organisation of microtubules. (**A**) Microtubule filaments are comprised of multiple dimeric complexes of α- and β-tubulin, assembled around a hollow core. Thirteen protofilaments assemble to form a microtubule. Microtubules are anchored at their minus ends at MTOCs, which is mediated by γ-tubulin. (**B**) Microtubules form dynamic networks in the cytoplasm which are stably anchored at MTOCs, including the centrosome and Golgi apparatus. Three-dimensional structural data: PDB ID tubulin dimer (1TUB).

Structurally, dimers of α- and β-tubulin polymerize to form microtubules, which are composed of 13 protofilaments assembled around a hollow core (Figure 1) [3].These filaments are subject to ongoing polymerisation and subsequent depolymerisation, which results in a protein network that is capable of undergoing rapid and continuous alterations in structure to serve the changing requirements of the cell (Figure 1).

The regulation of the microtubular cytoskeleton is mediated by post-translational modifications (PTMs) of constituent tubulin, along with microtubule associated proteins (MAPs) [4]. Microtubules are subject to numerous PTMs, including acetylation, phosphorylation, tyrosination, and palmitoylation, which induce profound effects on microtubule form and function. Microtubule associated PTMs can give rise to subpopulations of microtubules with specialized functions within the cell. For example, research demonstrates that distinct kinesin family motor proteins can identify and selectively interact with subpopulations of microtubules for preferential traffic towards specific microenvironmental domains [5]. Microtubule PTMs have also been demonstrated to control the spatial arrangement of cellular organelles. For example, detyrosinated microtubules sequester lysosomes and mediate their interactions with autophagosomes during autophagy [6]. Therefore, PTMs characterize distinct subgroups of microtubules that can be utilized by the cell for specific functions. Perhaps the most widely researched microtubule-associated PTM is acetylation, a modification of poorly understood functional significance, which enhances microtubule stability and modulates filament architecture [7].

A number of enzymes regulate the reversible acetylation of tubulin: The acetyltransferases ARD1-NAT1, ELP3, San, and αTAT1 [8,9,10,11,12,13], and the deacetylases histone deacetylase 6 (HDAC6) and SirT2 [14,15]. Tubulin acetylation occurs via the modification of the K40 residue of α-tubulin on the luminal surface of microtubules. In mammals and nematodes, these modifications are specifically dependent on αTAT1 [12,16,17], which is a member of the Gcn5-related N-acetyltransferase superfamily and a BBSome-associated protein [18]. The increased acetylation of microtubules is characteristic of stable filaments and it has been demonstrated to enhance interaction between microtubules and their associated motor proteins [19,20]. For example, the binding affinity of kinesin-1 for microtubules is enhanced when tubulin is hyperacetylated [19].

Non-motor MAPs provide the cell with a second level of microtubule regulation. The Tau family MAP proteins, which include Tau, MAP2, and MAP4, promote the assembly and stabilisation of microtubules, by enhancing longitudinal contacts within the filaments and protecting them from depolymerisation [21,22,23]. Tau family MAPs also competitively inhibit the binding of dynein and kinesin motor proteins to microtubules and, as such, are able to modulate their function as intracellular transport regulators [24,25,26]. Negative regulators of microtubule stability include the MAP stathmin, a protein that is capable of sequestering tubulin subunits and subsequently promoting the depolymerisation and shrinkage of microtubules [27,28]. The functions of stathmin have been specifically related to the regulation of the cell cycle, during which microtubule architecture undergoes dynamic alteration [29]. Efficient microtubule regulation is an essential prerequisite for adequate function of the protein filaments in numerous biological pathways.

While microtubules participate in a wide array of cellular processes, intracellular transport regulation is perhaps one of their most significant functions in numerous cell types. By forming dynamic tracks through the densely packed, cellular microenvironment, microtubules provide the fastest means of targeted traffic towards the perinuclear region, for a wide array of cargoes, including membranous organelles, proteins, and pathogens [30,31].

Cellular motor proteins represent a structurally and functionally diverse family of macromolecules that allow for the bidirectional transport of a variety of cargoes along cytoskeletal filaments. The motor proteins kinesin and dynein are essential for the transport-associated functions of microtubules [32]. Through direct interaction with microtubules, dynein motor proteins typically facilitate retrograde transport of biological cargo, such as endocytic vesicles, towards the cellular interior [33]. Kinesin motors typically regulate the transport of cargoes in an anterograde direction, towards the cellular periphery [34]. Several exceptions to this rule have been experimentally demonstrated. The C-kinesin family members KIFC2 and KIFC3 are able to participate in retrograde cargo transport [35], along with the kinesin-14 protein, KIFC1, which functions to maintain the localisation and architecture of the Golgi apparatus via retrograde movement [36].

Microtubules and their associated motor proteins require network-level, structural organisation into ordered arrays of filaments in order to allow for directed movement of any given cargo. The organisation of microtubules is co-ordinated at common positions throughout eukaryotic cells that are known as microtubule organising centres (MTOCs). MTOCs facilitate dynamic alterations in microtubular ‘tracks’ by anchoring microtubules at their minus ends and allowing for nucleation to proceed [37]. Nucleation, which represents the primary process that regulates spatial filament arrangement, occurs at the plus ends of microtubules and is an essential prerequisite for outward polymerisation towards the cellular periphery [38].

The most well established MTOC is the centrosome, which consists of a central pair of centrioles that are surrounded by pericentriolar material (PCM) [39]. In vertebrate cells, pericentrosomal Golgi membranes have also been identified as independent MTOCs with the ability to assemble and stabilise arrays of microtubules [40,41]. γ-tubulin, which is an important constituent of PCM, has been identified as a facilitator of microtubule nucleation [42,43]. MTOC-associated γ-tubulin also ensures stabilised anchoring and the subsequent organisation of microtubules [44]. While distinct bodies, such as the centrosome and Golgi, have been identified as MTOCs, numerous other centres for microtubule organisation are believed to exist within eukaryotic cells [45].

## 2. Microtubules in Influenza Virus Entry

A variety of animal viruses and bacteria, including adenoviruses, herpes, and influenza viruses, depend on microtubules [32,46,47,48,49]. Although the dynamic interactions between specific viruses and host-cytoskeletal proteins vary greatly, the microtubular network consistently provides a means of directed transport for invading pathogens. The microtubular cytoskeleton undergoes structural reorganisation following viral infections, demonstrating the tendency for invading pathogens to not only utilise, but also structurally alter, host-cell cytoskeletal networks during replication. The significant contribution of the microtubular network to the transport of influenza virus is perhaps primarily evidenced by the perinuclear location of MTOCs. The close association of MTOCs with the nucleus identifies microtubular filaments as a direct route for influenza viruses towards the site of viral genome replication [32]. In agreement with these observations, numerous studies have demonstrated diminished viral infection following microtubule disruption in vitro [50]. Microtubules facilitate essential virus-host interactions during the sub-cellular journey of influenza, in particular during viral entry and egress, in addition to forming a physical bridge between viruses and their replication and assembly sites.

In eukaryotic cells, endocytosis facilitates the uptake of external cargo. A wide variety of materials, including membrane-associated receptor-ligand complexes, proteins, lipids, and intracellular pathogens, are internalised via endocytosis [51,52,53]. The cellular machinery for endocytosis consists of a network of membranous compartments, which provide specialised microenvironments for targeted cargo transport [51]. Following uptake into the cell, endocytosed cargo is contained within an early endosome (EE), a specialised cellular vesicle that is characterised by the presence of Rab5, a small GTPase that regulates several aspects of endosome maturation [51]. As endocytosis progresses, Rab5-dependent endosome maturation occurs, inducing a Rab switchover, such that late endosomes (LEs) are primarily associated with Rab7 [51]. Endosome maturation is essential for efficient downstream cargo trafficking.

Biological cargo that is transported via the endocytic pathway has two major fates; recycling back to the plasma membrane or traffic towards the lysosome-associated degradative pathway [51,52]. While the majority of internalised cargo is recycled back to the plasma membrane, a defined subset of material, often including invading pathogens, is targeted towards lysosomes [51]. For a number of viruses, including influenza, the endocytic pathway is exploited for the purposes of priming, which occurs during subcellular trafficking and targeted transport to the nucleus, the site of viral genome replication. Following uptake into EEs, IAVs traffic along the endocytic pathway to the perinuclear region, before escaping the endosomal compartment via low-pH induced fusion at LEs.

**Figure 2:**
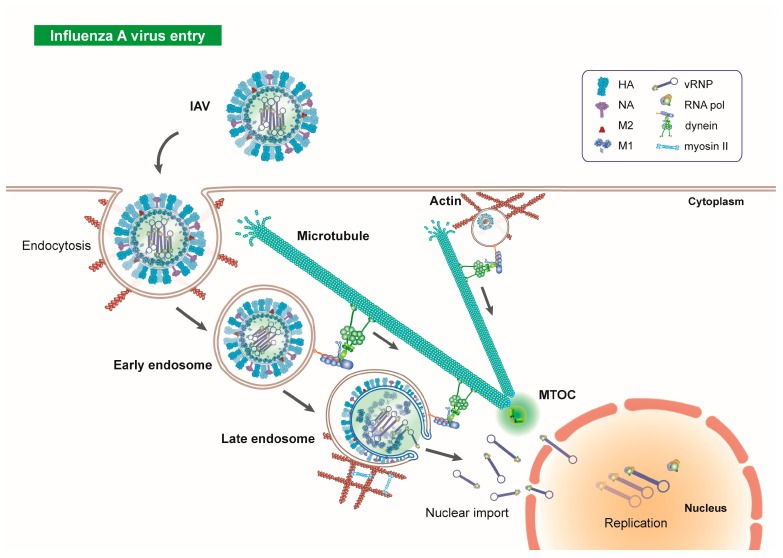
Influenza A virus endocytosis and early trafficking through the cell. IAV is a single-stranded negative sense RNA virus, belonging to the Orthomyxoviridae family. Viral particles are composed of an outer envelope containing the glycoproteins hemagglutinin (HA) and neuraminidase (NA) and M2 ion channels. An M1 shell constitutes the viral shell, within which are 8 viral gene segments each in association with nucleoprotein (NP) and an RNA polymerase. The influenza polymerase itself is formed from three subunits; PA, PB1 and PB2. Following attachment of IAV to permissive cells via sialylated cell-surface receptors, the virus is endocytosed via clathrin-mediated endocytosis and macropinocytosis. After initial association with the actin-myosin network, early endosomes containing IAV virions interact with microtubules via dynein motor proteins for retrograde traffic towards the MTOC, in close proximity to the cellular nucleus. Upon reaching the perinuclear region, IAVs undergo low-pH mediated fusion with the late endosomal membrane. M1 shell uncoating is dependent on microtubules, actin, and the motors dynein and myosin II. Release of vRNPs into the cytosol and uptake into the nucleus precedes viral genome replication. Three-dimensional structural data: PDB ID HA (2IBX); NA (6CRD); M2 (3BKD); M1 (1EA3) [65].

The cellular cytoskeleton and its composite microtubules form fundamental components of the endocytic machinery, which are exploited by invading IAV particles (Figure 2). While some viruses, including herpes, polyoma, adeno, and adeno-associated viruses, are able to directly interact with microtubular motor proteins for transport [54,55,56,57], others, including IAV, rely on endocytic vesicles for interaction with and traffic along microtubules [58]. Within the cellular periphery, endosomes and their cargo interact with actin filaments [59,60], which, together with their associated myosin motors, facilitate the short, back and forth motion of EEs. As endosomes move towards the cellular interior, retrograde transport becomes dependent upon microtubules and their associated dynein motors [61,62,63]. The endocytic machinery requires a means of sorting, such that directed transport of macromolecules to their appropriate cellular compartments can occur, since the fate of endocytosed cargo varies. Endosome sorting is largely dependent on sorting nexins (SNXs), proteins, which interact with microtubule associated motors and mediate endosome-microtubule interactions, subcellular trafficking, and localisation [64]. Therefore, endosome sorting is dependent on intact microtubules [61], which serve as essential scaffold proteins during this process.

The importance of intact microtubules for transport of EEs towards the nucleus is evidenced not only by the association between microtubule-linked motors and endocytic vesicles, but also by microtubule inhibition studies. The depolymerisation of the microtubular network induces the dispersal of mature endosomes throughout the cytoplasm [66]. Intact microtubules promote IAV entry into cells [58,60,67,68,69,70,71]. Real-time fluorescent microscopy studies of individual influenza viruses, along with quantum-dot based viral tracking techniques, have provided evidence that endosome-contained IAVs utilise classical endocytic pathways and microtubules during transit through the cytoplasm (Figure 1) [58,60,72]. Specifically, IAVs induce the formation of clathrin coated pits (CCPs) for uptake into endocytic vesicles, and also use caveolin-independent endocytosis and pinocytic uptake mechanisms [69,73].

Following endocytosis, EE-contained IAV particles undergo a three-stage transport process to the perinuclear region. In the cellular periphery, endosomes that contain IAVs interact with actin microfilaments and undergo slow restricted movements, close to the plasma membrane [58,72]. Closer to the cellular interior, IAV-containing endosomes interact with microtubule-associated dynein motors for rapid, retrograde transport towards the nucleus [72] (Figure 1) and they undergo bidirectional movements along microtubules at the nuclear periphery [58].

In addition to facilitating targeted transport of IAVs towards the cellular interior, endocytic vesicles also provide specialised sub-cellular microenvironments that allow for optimal replication of influenza. Endocytic vesicles are separated from the surrounding cytosol by a phospholipid bilayer. While cytosolic pH is typically maintained at around 7.4 [74], ATP-dependent proton pumps in the endosomal membrane, known as vaculoar-ATPases (v-ATPases), modulate the intraluminal acidity of endosomes [75,76], within the range of pH 6.5–4.5. Acidic pH levels within endosomes optimise the catalytic activity of numerous enzymes, which function to sort, process, and degrade a wide variety of protein cargoes under physiological conditions [74,77,78]. For influenza viruses, the characteristically progressive acidification of the endosomal compartment during movement towards the nucleus represents an essential regulator of downstream viral replication. Specifically, IAV is dependent on low-pH endosomes for efficient uncoating, which promotes successful genome release into the cytosol [79,80,81,82].

The contribution of microtubules to endosome maturation and acidification has been experimentally demonstrated via the disruption of the cytoskeletal network with depolymerizing agents and motor protein inhibitors. Microtubule depolymerisation as well as dynein motor disruption demonstrate a consistent ability to delay the maturation of endocytic vesicles [83]. While these consequences do not prevent the early acidification of endosomes to pHs of around 6 [84], slowed maturation might delay cargo processing and prevent intraluminal pH dropping to lower levels [66,84]. For influenza viruses, intact microtubules significantly contribute to replicative success, with viral infection being halved in vitro following treatment with nocodazole [70,85].

## 3. HDACs, Microtubules and Endocytosis

During viral endocytosis, the activity of HDAC enzymes exerts profound effects on microtubules and influenza replication. While best known for their functions in chromatin remodelling and control of gene expression [86], HDACs also deacetylate multiple non-histone proteins, including microtubules [87]. Therefore, HDACs represent important intermediates between influenza viruses, microtubules, and endosome maturation.

HDACs can be divided into three subclasses; Class I, II, and III [88]. Class I HDACs (HDAC1, 2, 3, 8) are able to modulate the productive entry of IAV by influencing microtubule architecture, the maturation of endosomes, and the downstream endocytic pathway. In HDAC8-depleted cells, centrioles separate, while microtubules lose both organisation and the ability to form asters upon regrowth. The Golgi, LEs, and lysosomes (LYs) demonstrate dispersal throughout the cytoplasm and lack proper motility. As a result, IAV infection is blocked in HDAC8 depleted cells. Interestingly, the opposite phenotype is observed in cells depleted of HDAC1, while microtubule networks maintain normal architecture, centripetal accumulation of the Golgi, and LE/LYs leads to enhanced IAV infection. Collectively, these results demonstrate that class I HDACs participate in the regulation of the endocytic pathway, especially the pathway from EEs to LYs [70]. By modulating centrosome architecture and downstream microtubule organisation, HDACs 1 and 8 directly influence endosome trafficking and IAV transport in infected cells. Therefore, influenza-HDAC interplay is essential for efficient replication and identifies microtubules as downstream effector proteins in these cellular pathways.

In summary, IAVs depend on the microtubule network during the endosomal stage of viral entry. Primarily, microtubules facilitate the perinuclear transport of invading virions during endocytosis and ensure the optimal targeting of influenza viruses towards their replication site. In addition, microtubules support endosome maturation and, therefore, optimisation of the microenvironment needed for viral uncoating. While endosome acidification cannot be completely disrupted by microtubule inhibition, the filaments still serve as important accessory proteins for optimal maturation of endocytic vesicles, as shown by research demonstrating alterations in influenza infection upon changes in HDAC1 and 8 expression.

## 4. Influenza Virus Priming in Endosomes

Uncoating represents an essential stage in the entry of influenza and an important pre-requisite to the establishment of infection. In fact, amantadine, a viral M2 inhibitor that blocks uncoating, was once widely utilised to control human influenza infections. IAV uncoating involves two steps: priming and physical disassembly of the viral M1 shell. Priming takes place within endocytic vesicles and it is determined by acidification of the viral core microenvironment, which is initiated in EEs, where pH levels reach between 6.5 and 6.0. Gradual acidification of the endosome interior triggers priming via the M2 ion channels present on the viral membrane [81,82,89]. As pH drops, M2 channels open, allowing for the influx of protons and K^+^ ions into the viral core [79,81]. In addition, as a consequence of gradual pH reduction, conformational changes are initiated in the viral fusion glycoprotein hemagglutinin (HA), which optimise the viral envelope for fusion [90,91]. In essence, these modifications act to soften the viral core in preparation for genome release. Specifically, protons weaken the interactions between HA-M1, M1-M1, and M1-vRNP, while the K^+^ ions promote the solubilisation of vRNP complexes. Within LEs, where the microenvironmental pH reaches less than 5.5, irreversible changes take place within the viral core, which are needed for the efficient dissociation of the viral shell from vRNP complexes [81,92].

Viral priming precedes HA-mediated fusion with the endosomal membrane, physical disassembly of the capsid, and subsequent genome release into the cytoplasm. The importance of microtubules in progressive endosome acidification has been previously demonstrated [66]. Mathematical modelling further suggests that coordinated endosome acidification and microtubular transport serve as limiting factors during influenza virus infection [93]. Viral transport by microtubules to a perinuclear fusion site represents an optimal condition for successful infection since premature exposure of invading virions to the cytosol can enhance vRNP degradation [93]. Thus, the trafficking of IAVs along microtubules within endocytic vesicles serves not only to protect incoming viruses from immune detection [94], but it is also optimal for perinuclear fusion, at sites where LEs concentrate. This indicates that, although a functional microtubule network is dispensable for sufficient viral priming, disrupted endosome trafficking can limit influenza infectivity.

## 5. Influenza Virus Uncoating and Aggresome Processing

Microtubules are important mediators of IAV shell disassembly. These functions are linked to the roles of microtubules and their associated motor proteins, within misfolded protein-containing membraneless organelles, called aggresomes [95]. Aggresomes typically mediate the disaggregation of misfolded proteins via the actions of microtubule-linked dynein and actin-linked actomyosin motors. Aggresome formation occurs when the cellular capacity for proteasomal protein degradation is exceeded, which leads to the accumulation of ubiquitin conjugated, misfolded protein aggregates in the cytoplasm [96]. In addition to its aforementioned role in mediating tubulin deacetylation, cytosolic HDAC6 is a key component of the aggresome processing machinery, which interacts with polyubiquitin chains via its zinc-finger ubiquitin binding domain (ZnF-UBP) [97]. HDAC6 essentially serves as an adaptor within aggresomes, linking target proteins to molecular motors, including dynein and myosin II, for assembly at the MTOC and subsequent disaggregation [95,97,98,99]. Aggresome formation depends on the microtubular cytoskeleton and its associated dynein motor proteins [95,100,101]. Aggresomes accumulate at MTOCs in close proximity to centrosomes, which indicates that misfolded proteins undergo directed movement along microtubules [100]. It has been subsequently suggested that centrosomes themselves may form scaffold structures for aggresome complexes, which allow for successful interactions between multiple protein components, including cellular ubiquitin, Hsp70, Hsp90, and HDAC6 [95,102]. In agreement with this essential role for microtubules as mediators of aggresome formation, treatment of cells with microtubule depolymerising agents and other inhibitory compounds completely blocks the formation of aggresomes, as detectable by microscopy [100,101].

Aggresomes and their constituent proteins, HDAC6, unanchored ubiquitin, and molecular motors, are exploited by invading influenza viruses for the completion of uncoating via core disassembly, breakdown of the viral M1 shell, and debundling of vRNPs [95,97,100,103]. Therefore, a functional microtubule network represents an important prerequisite for the assembly of the cellular machinery needed for IAV shell breakdown during uncoating. In addition to their roles in enabling aggresome assembly, microtubules also form an important functional constituent of the aggresome via their interactions with dynein motor proteins. Cytoplasmic dynein and its cofactor dynactin act in concert with microtubules during protein processing within aggresomes. Specifically, the p50 subunit of dynactin, which is also known as dynamitin, mediates the attachment of dynein to target proteins [96]. HDAC6 contains a dynein-binding region between its two catalytic domains in the N-terminal region [95,104]. The removal of the HDAC6 dynein-binding region reduces IAV uncoating by 30%, whereas mutating the HDAC6 ZnF-UBP reduces it by 80% in comparison to control. This indicates that factors recruited to HDAC6 following ubiquitin chain binding to the ZnF (e.g., myosin II) must synergise with dynein to complete uncoating [97].

Opposing physical forces that are generated by motor proteins and their associated cytoskeletal scaffolds promote the disassembly of misfolded proteins and viral capsids, including that of adenovirus [105,106]. IAV also exploits the shearing force that is provided by motor proteins by hijacking the aggresome pathway. The virus enables this by encapsidating unanchored ubiquitin chains into the virion during replication in producer cells [97]. Following the priming and fusion of IAVs with LE membranes, the ubiquitin chains expose to the cytosol, recruit HDAC6, and activate aggresome processing [97]. Microtubule- and actin-dependent motor proteins subsequently facilitate the physical breakdown of the viral shell and promote genome release into the cytoplasm. HDAC6-mediated shell disassembly is followed by vRNP uncoating and debundling by karyopherin-β2 (Kap β2), also known as transportin-1 (TNPO1), which is a member of the importin-β family of nuclear transport receptors (NTRs) [103].

## 6. vRNP Nuclear Import

In order to access the nucleus, vRNPs must traverse the nuclear envelope, a double layered membrane that selectively excludes macromolecules too large to passively diffuse through nuclear pores [107]. The shuttling of macromolecules, including proteins, RNA, and some viruses, between the cytoplasm and nucleoplasm, is mediated by nuclear pore complexes (NPCs), large multi-protein structures that span the nuclear envelope and form aqueous channels for cargo trafficking [107,108,109].

Nuclear import and export are tightly regulated processes that are primarily controlled by NTRs. NTRs are members of the Karyopherin superfamily and they include importin, exportin, and transportin proteins [110,111,112]. Cargo recognition and interaction with NTRs is primarily controlled by the presence of nuclear localisation signals (NLSs) or nuclear export signals (NESs) in target proteins. Classical NLSs comprise short, basic amino acid sequences (e.g., KKKRK) [113], which are specifically recognised and bound by NTRs [114,115]. The most widely researched NTR is importin-α, a nuclear import receptor, which, when bound to importin-β, traffics a wide variety of cargo across the nuclear envelope [111]. These shuttling processes are active and as such, rely on ATP hydrolysis and secondary regulation by Ran GTPases that control the directionality of transport through NPCs [116,117,118].

The function of NTRs and their regulators rely on their ability to freely diffuse within cellular compartments and interact with appropriate binding partners. Competitive binding between nuclear transport regulators, including importin-α, importin-β and NTF2, and microtubules, may serve as an important regulatory mechanism controlling downstream nuclear import processes [119]. For example, the sequestering of NTRs as well as their regulatory proteins by immobile microtubules has been shown to negatively regulate the nuclear import of several proteins [120,121,122].

Influenza viral proteins express a wide variety of NLSs and NESs, which allow for them to interact with importins and exportins for translocation across the nuclear envelope [111,123,124]. Numerous influenza viral proteins, including NS1, PB1, PB2, PA, M1, and NP, have been identified to contain NLS and NESs [111]. Viral NP which contains one unconventional NLS, NLS1, and one bipartite NLS, NLS2, (^3^TKGTKRSYEQM^13^/^198^KGINDRNFWRGENGRRTR^216^, respectively), has been subject to mutational analyses demonstrating that NLS2 is essential in viral genome replication [124,125]. In contrast, NLS1 more significantly contributes to the nuclear accumulation of viral proteins [126]. The IAV M1 protein contains an NLS (^101^RKLKR^105^) and an NES (^59^ILGFVFTLTV^68^), which are required for viral genome replication [127,128]. In addition, M1 has an acid exposed “PY-less” PY-NLS (^18^GPLKAEIAQR^27^), which recruits Kapβ2 for vRNP uncoating and debundling [103].

For a variety of proteins, it is generally accepted that nuclear import processes are independent of cytoskeletal regulation [129]. However, research has demonstrated that the microtubular cytoskeleton might play a significant part in the nuclear import of several cancer associated proteins. Protein cargoes, including parathyroid like hormone protein (PTHrP), p53, and retinoblastoma (Rb), have been shown to depend on functional microtubules for nuclear accumulation [129,130,131,132].

The nuclear import of several viruses, including human immunodeficiency virus (HIV), herpes simplex virus type 1 (HSV-1) [54,133], and rabies virus [134], have been studied with regards to microtubules [129]. For several viruses, microtubules play an important part in determining viral protein accumulation. Rabies virus, herpesviruses, and HIV translocate to the NPC via interactions of viral capsid proteins with dynein and kinesin motors and their associated microtubules [133,135,136]. Inner tegument viral proteins of HSV-1 directly interact with dynein and kinesin-1 for microtubule mediated bidirectional transport through the cytoplasm [133,137]. HIV interacts with microtubule-linked dynein via the BICD2 (Protein bicaudal D homolog 2) adaptor protein for capsid transport towards the nucleus [138]. In addition, the nuclear accumulation of HIV relies on FEZ1 (Fasciculation and elongation protein zeta 1), which is a kinesin-1 associated adapter protein [139]. The nuclear accumulation of rabies virus phosphoprotein (P-protein) is significantly enhanced by its dynein binding sequence, and nuclear import of P-protein depends on intact microtubules [134]. In contrast, the nuclear import of influenza vRNPs is independent of microtubules [70,82,140].

In mammalian cells, an indirect link has been identified between the nuclear import receptors and microtubules. TPX2, which is a regulator of microtubule nucleation that directly interacts with the filaments, is itself regulated by importin-α [141]. The dependence of importin-α on the microtubular network for intracellular trafficking has not been studied in detail. However, the associations between importin-α and microtubules, both direct and indirect, suggest that the microtubular cytoskeleton could contribute to the localisation of NTRs and their subsequent interactions with IAV. Further exploration of the contributions of microtubules to the perinuclear accumulation of nuclear import regulators might reveal novel mechanisms of cytoskeletal exploitation by influenza during this stage of the viral life cycle.

## 7. Microtubules in Influenza Virus Egress

**Figure 3:**
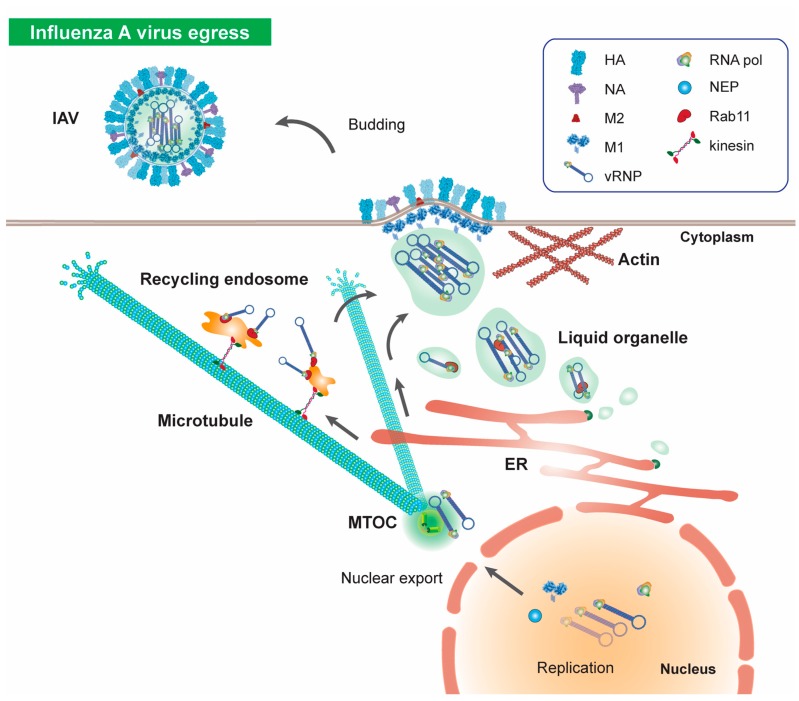
Influenza A virus egress. Following replication of viral RNA, newly synthesised vRNPs are exported from the nucleus and accumulate at the MTOC, before trafficking towards the cellular periphery in a microtubule dependent manner for assembly and budding. Influenza viruses utilise components of the endocytic recycling and secretory pathways for apical transport; associations between Rab11-positive recycling endocytic vesicles and influenza viruses allow viral traffic along microtubules. In addition, vRNPs induce formation of liquid organelles which associate with Rab11 for vRNP traffic via the secretory pathway. Following microtubule-dependent anterograde traffic to the cellular periphery, vRNPs assemble to form new virions and bud from the cell surface to trigger secondary infection in permissive cells. Three-dimensional structural data: PDB ID HA (2IBX); NA (6CRD); M2 (3BKD); M1 (1EA3) [65].

Following genome replication, influenza vRNPs must be exported to the cytoplasm and egress to the plasma membrane for virion assembly and budding (Figure 3). The nuclear export of vRNPs requires the assembly of a nuclear export complex containing vRNPs, M1, and nuclear export protein (NEP), which contains two NESs. This complex mediates the association of exportin1/XPO1/CRM1 with vRNPs and translocation of viral proteins from the nucleus to the cytoplasm [142,143,144,145,146]. The enlargement of nuclear pores via increased activation of caspase 3 is characteristic of influenza infected cells and serves to enhance vRNP export [147].

Immediately following nuclear export, vRNPs accumulate at the MTOC, where they demonstrate intermittent, saltatory motion that is characteristic of microtubule-based motility [85,148,149,150,151,152]. MTOC accumulation is dependent on microtubules, as well as YB-1 (Y-box binding protein 1) and HRB (HIV reverse binding protein) [153,154]. MTOC-accumulated vRNPs induce dynamic alterations in the cellular microenvironment to build an optimal platform for protein trafficking; by recruiting and activating Rab11 and YB-1, influenza viruses induce centrosome maturation in infected cells, which leads to cholesterol enrichment along with microtubule anchoring and network remodelling [154]. These cellular changes facilitate interactions between the PB2 subunit of the viral polymerase and Rab11 [148,154]. The interactions of vRNPs with Rab11 play an essential part in their outward trafficking to the plasma membrane via the endocytic recycling and secretory pathways (Figure 3).

The endocytic recycling pathway is essential for anterograde cargo transport and it ensures that proteins and lipids are appropriately trafficked for cellular secretion or incorporation into the plasma membrane [155,156]. The endocytic recycling compartment (ERC) comprises a collection of juxtanuclear, tubular organelles which form via the maturation of EEs and are defined at a molecular level by the presence of Rab11 [77,157]. In uninfected cells, Rab11-GTP regulates cargo transport by interacting with cytoplasmic motor and tethering proteins, which mediate transport to, and docking of, Rab11-positive vesicles with the plasma membrane [158].

Positive- and negative-sense RNA viruses, such as paramyxovirus, retrovirus, and orthomyxo viruses, including influenza, use Rab11-positive vesicles for egress towards the plasma membrane (Figure 3) [148,152,158,159,160,161]. Since Rab11 forms a key component of the ERC these observations suggest a model of influenza egress whereby newly synthesised viral proteins utilise Rab11 for docking with recycling endosomes in the vicinity of the MTOC [148,149]. Although this model of egress is supported by several studies demonstrating that Rab11 is essential for vRNP trafficking to the plasma membrane [148,153,160], emerging evidence suggests that alternative routes for vRNP transport exist within infected cells (Figure 3).

Influenza infection can induce dynamic changes in the sub-cellular localisation of Rab11 [154,155,162]. Mechanistically, vRNPs interfere with the binding of Rab11 to its effector proteins, FIPs (Rab11-family interacting proteins) [155], which redistributes Rab11 to the ER and impairs its GTPase function [162]. These changes are likely to impair the ERC pathway in influenza infected cells, with resultant sub-optimal trafficking of vRNPs, should recycling endosomes be the primary means of viral egress.

Influenza infection has also been associated with global remodelling of the ER and the formation of virus-associated organelles [152,162]. Newly synthesised vRNPs form distinct cytoplasmic inclusions within liquid organelles, which predominantly utilise the ER and secretory pathway for transport towards the cell periphery [152]. HDAC6 regulates cellular phase (liquid-liquid or liquid-solid) separation via the deacetylation of intrinsically disordered regions in substrate proteins, promoting the formation of liquid-phase organelles [163]. Since egress of influenza is dependent on formation of liquid-phase membraneless organelles [152], the regulatory functions of HDAC6 are potentially relevant for vRNP peripheral transport.

The secretory pathway is an alternative means of peripheral transport for macromolecules and it encompasses the rough-ER, the Golgi, and post-Golgi carrier vesicles [164,165]. The organelles of the secretory pathway respond to a wide array of exogenous and endogenous stimuli and maintain a distinct, cell-type specific organisation [165,166]. The secretory pathway provides a second, alternative route to the plasma membrane for newly synthesised vRNPs. Influenza proteins must interact with membranous compartments, including the ER and Golgi, to utilise the secretory pathway for anterograde transport. In vertebrate cells, the pericentrosomal location and structural integrity of the Golgi are both maintained by functional microtubules [167]. The Golgi itself is an established MTOC, with the ability to independently generate organised arrays of microtubule filaments [40,41]. The negative ends of microtubules are also able to dissociate from centrosomes and interact with the Golgi membrane [167]. Golgi-associated MTOC activity is essential for its role in the secretory pathway and it provides a mechanism of transport for viral proteins. M2 and HA, which utilise the Golgi for interaction with the secretory pathway [168,169,170], are therefore dependent on microtubules for the maintenance of Golgi architecture as well as downstream trafficking.

While the contributions of the endocytic recycling and secretory pathways to influenza virus egress and budding are still a topic of debate, it is clear that Rab11 and microtubules are necessary for optimal vRNP trafficking post-nuclear export. Microtubules and their associated organising centres represent common components of the cellular machinery that is involved in both the endocytic recycling and secretory pathways. As such, the disruption of microtubule architecture with depolymerising agents reduces viral budding [85,149,171], and disperses punctate, cytoplasmic vRNP signals [160]. Blocking the actin-myosin network interferes with budding and reduces viral titers [172]. Influenza viruses require microtubules for optimal egress; while actively participating in the transport of ERC-associated cargo, microtubules also support the architecture of the cellular machinery that is necessary for a functional secretory pathway.

During viral genome replication and egress, HDAC6 once again serves as an important host-cell regulatory factor for influenza. HDAC6 activity has inhibitory effects during IAV assembly and egress and thus must be deactivated for optimal viral replication [97,173,174,175]. HDAC6 restricts viral replication by deacetylating the PA subunit of the viral RNA polymerase [176] and Lys909 of retinoic-acid inducible gene I (RIG-I). These changes lead to RIG-I oligomerisation, viral RNA sensing, and activation of the mitochondrial antiviral signalling protein (MAVS)-IRF3-NF-kB and IFN-β [177]. By interacting with microtubules via β-tubulin binding and deacetylating α-tubulin, HDAC6 is also able to destabilise the microtubular cytoskeleton [175] and exert a negative effect on IAV egress [173]. During IAV infection, viral induced degradation of HDAC6 via caspase 3 promotes α-tubulin acetylation and microtubule stability. These virally induced cellular changes also prevent premature uncoating, since active HDAC6 mediates the physical disassembly of the M1 shell of IAV [98,173,174,178].

Therefore, the roles of HDAC6 as an antiviral can be attributed in part, to its function as a regulator of tubulin acetylation. Microtubules represent a common intermediate between HDAC6 and invading influenza viruses, at multiple stages of the viral life cycle and viral induced degradation of HDAC6 serves an essential purpose during egress; the maintenance of a stable microtubule network for efficient trafficking of newly synthesised vRNPs.

## 8. Conclusions

Microtubules are essential regulators of an array of processes, with fundamental roles in controlling cell morphology, motility, and intracellular transport. Microtubule networks are exploited by invading pathogens, which are reliant on the cytoskeleton at multiple stages of infection. For influenza viruses, microtubules facilitate intracellular transportation at multiple stages during the viral life cycle. Microtubules and their associated proteins also mediate physical disassembly of the viral shell and genome release into the cytoplasm. In addition, influenza infection results in structural modification of the microtubule network, often via viral impacts on lysine deacetylases, HDACs. These dynamic alterations in cytoskeletal architecture often facilitate replicative success. Characterising the mechanistic links between a functional microtubule network and successful viral replication provides significant insight into virus-host interactions and enhances understanding of the dynamic alterations in host-cell biology as a consequence of influenza infection.
